# A fine line between attack and care

**DOI:** 10.7554/eLife.103351

**Published:** 2024-10-16

**Authors:** Nicole Rigney

**Affiliations:** 1 Social Neuroscience Laboratory, University of California, Los Angeles Los Angeles United States

**Keywords:** PVN, paraventricular nucleus, mPFC, medial prefrontal cortex, oxytocin, infanticide, mandarin voles, Other

## Abstract

Oxytocin neuron projections from two brain regions involved in parental care regulate both parental care and infanticidal behaviors in virgin mandarin voles.

**Related research article** Li L, Li Y, Huang C, Hou W, Lv Z, Zhang L, Qu Y, Sun Y, Huang K, Han X, He Z, Tai F. 2024. PVN-mPFC OT projections modulates pup-directed pup care or attacking in virgin mandarin voles. *eLife* **12**:RP96543. doi: 10.7554/eLife.96543.

Oxytocin, commonly known as the 'love hormone', plays a vital role in social functioning, from pair bonding to parental care. It is well known for establishing early attachment between mothers and their infants as well as bonds between romantic partners.

Oxytocin is produced in the paraventricular nucleus of the hypothalamus, which transmits signals to regions in the brain rich in oxytocin receptors, such as the medial prefrontal cortex (mPFC). This area is involved in higher-order cognitive functions, including decision-making, behavioral flexibility and parental care (Froemke and Young, 2021; Rigney et al., 2022; Rilling and Sanfey, 2011; Dulac et al., 2014). Previous research has shown that increased oxytocin levels in the paraventricular nucleus are linked to parental care behavior (He et al., 2021). For example, oxytocin released from the paraventricular nucleus in mice enhances the importance of pup vocalizations (Carcea et al., 2021; Schiavo et al., 2020). Likewise, human studies have shown that activity in the mPFC increases when mothers hear their infants cry (Lorberbaum et al., 2002). This suggests that the projections of oxytocin neurons connecting the paraventricular nucleus to the mPFC may be involved in controlling parental care.

Parental care is critical for offspring survival in many species and the transition to parenthood is known to alter parental care motivation. Unlike many commonly studied laboratory rodents, both male and female mandarin voles are involved in caring for offspring (Young et al., 1998). However, both virgin males and females of this species can show aggression – often leading to infanticide – towards unrelated pups. This makes them an ideal model organism for studying the neurological basis of these contrasting behaviors. However, we do not fully understand how oxytocin influences the balance between care and infanticide. Now, in eLife, Fadao Tai, Zhixiong He and colleagues at Shaanxi Normal University in China – including Lu Li as first author – report new insights into the neural circuits underlying parental behavior and infanticide in mandarin voles (Li et al., 2024).

Li et al. used a combination of techniques, including immunohistochemistry, optogenetics and injecting oxytocin into the abdominal area, to reveal the neural mechanisms underlying parental care and infanticide (Figure 1). Activating oxytocin neurons in the paraventricular nucleus using optogenetic techniques reduced the time it took for males to approach and retrieve pups, a measurement of pup-directed behavior. However, this activation had no effect on females. Activating the same neurons in voles of both sexes showing infanticidal behaviors lowered their infanticidal tendencies, while inhibiting oxytocin neurons in this brain area promoted infanticide. This suggests that oxytocin-expressing neurons in the paraventricular nucleus can promote parental care and inhibit infanticide (Figure 1).

**Figure 1.**
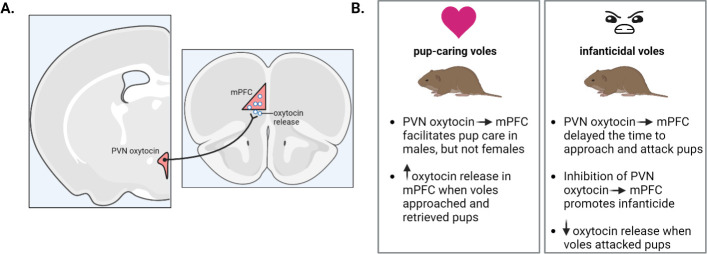
Oxytocin’s influence on parental behaviors in mandarin voles. (**A**) Oxytocin neuronal projections (black line) running from the paraventricular nucleus (pink) of the hypothalamus (left) to the medial prefrontal cortex (pink triangle; right) releases oxytocin (white circles) (B) Stimulating these oxytocin projections in pup-caring voles increased pup care in male but not female voles (left). Oxytocin release increased in both males and females when caring for pups but decreased during attacks. In voles showing infanticidal behavior (right), stimulating the oxytocin projections delayed infanticidal attacks while inhibiting the neurons promoted infanticide in both sexes. PVN: paraventricular nucleus; mPFC: medial prefrontal cortex. This figure was created with BioRender.com.

Similarly, stimulating oxytocin projections from the paraventricular nucleus to the mPFC increased pup care behavior in males, but not females, while inhibiting these projections promoted infanticidal behavior in both sexes. Using a fluorescent sensor to detect oxytocin revealed that its release increased in the mPFC of both male and female voles when they exhibited parental care behaviors, such as approaching and retrieving pups, but decreased in both sexes when voles attacked pups. Next, Li et al. administered oxytocin into the abdominal cavity (a method that could possibly translate to a clinical application) and observed the same changes in behavior when oxytocin was applied in this way.

The different responses of males and females across various experiments suggest additional, potentially sex-specific mechanisms might be involved in regulating parental care. Previous work has shown that male mice without the oxytocin or oxytocin receptor genes have trouble picking up and moving their pups. This problem is less noticeable in female mice (Inada et al., 2022). This phenomenon could stem from multiple neural systems that drive maternal caregiving behaviors in females. These backup mechanisms would ensure that maternal care remains robust even if oxytocin signaling is disrupted.

Another explanation could be that females tend to have a higher neural oxytocin activity, a greater number of oxytocin neurons, more extensive axon projections and distinct receptor expression patterns (Häussler et al., 1990; Insel et al., 1991; Uhl-Bronner et al., 2005). This may limit the impact of oxytocin neuron manipulation on female pup care behaviors, as they could already function close to their maximum.

Overall, Li et al. revealed that oxytocin projections from the paraventricular nucleus to the mPFC regulate pup care and infanticidal behaviors in virgin mandarin voles. Collectively, oxytocin appears to act as a switch capable of promoting nurturing as well as aggressive responses toward pups, depending on the context and the individual. The findings open new avenues for exploring other oxytocin and neurotransmitter circuits that may influence these parental and aggressive behaviors, and the reasons for the observed sex differences.
